# Navigating challenges in human pluripotent stem cell-derived islet therapy for type 1 diabetes

**DOI:** 10.3389/fimmu.2025.1625439

**Published:** 2025-08-04

**Authors:** Mohammed Usama, Ying Deng, Yiran Chen, Théa Milland, Mohan Malleshaiah, Yasaman Aghazadeh

**Affiliations:** ^1^ Institute de Recherches Cliniques de Montréal (IRCM), Montréal, QC, Canada; ^2^ Division of Clinical and Translational Research, Department of Medicine, McGill University, Montréal, QC, Canada; ^3^ Department of Medicine, University of Montréal, Montréal, QC, Canada; ^4^ Department of Anatomy and Cell Biology, McGill University, Montréal, QC, Canada; ^5^ Department of Biochemistry and Molecular Medicine, University of Montréal, Montréal, QC, Canada

**Keywords:** beta cells, islets, transplantation, human pluripotent stem cells, vascularization, immune cells, type 1 diabetes, immunosuppression

## Abstract

In the past two decades, several tissues have been generated from the differentiation of human pluripotent stem cells (hPSCs) to model development or disease, and for use in drug testing and cell replacement therapies. A frontliner of hPSC-derived tissues used in cell replacement therapies are the pancreatic cells, which have entered multiple clinical trials since 2014 for the treatment of type 1 diabetes (T1D). Despite challenges in early trials, the detection of endogenous C-peptide in recipients was encouraging. The results and challenges of these trials inspired new areas of research, leading to incremental advances in cell differentiation and delivery technologies, and a deeper understanding of the transplantation microenvironment to enhance therapeutic efficacy and longevity. Reports from the most recent trials demonstrated success in reducing or eliminating exogenous insulin administration for people with T1D, increasing hope for a cure for T1D via regenerative medicine. Recent efforts can be broadly categorized into: (1) improving the cell product as surrogates of native beta cells, (2) promoting engraftment post-transplant to support cell survival, integration into the host, and endocrine function, and (3) developing immunomodulation strategies to reduce or circumvent immunosuppression regimen. In this review, we discuss recent and emerging advances in these three areas and the potential, risk, and scalability of experimental models to the clinic.

## 1 Introduction

Type 1 diabetes is an autoimmune disease characterized by the gradual depletion of beta cell mass, resulting in insufficient insulin secretion to maintain normal blood glucose levels. While the discovery of insulin has changed T1D from a deadly disease to a manageable one, life-long insulin administration is burdensome, lowers quality of life, and is not as effective as endogenous insulin in regulating blood glucose and time in range (1). The isolation of donor islets and infusion into the portal vein has demonstrated that cell replacement therapy can restore endogenous insulin secretion to significantly improve glycemic control and the quality of life of the recipients (**Table 1**) (8). Human pluripotent stem cells (hPSC) derived beta cells have also shown great promise in secreting endogenous insulin after transplantation in research setting and in clinical trials (**Table 1**) (2, 5). The hPSCs can be differentiated via a robust 4-stage protocol to pancreatic endodermal cells (PECs), which are multi-potent progenitors of the pancreas and can continue to differentiate when transplanted *in vivo* or maintained *in vitro* for two additional differentiation stages (stages 5 and 6) to islet-like clusters (SC-islets), which contain beta-like cells (9). The PECs, first developed in 2006–2008 entered clinical trials in 2014, in which they were derived from the human embryonic stem cell (hESC) line CyT49, encapsulated in an immune cell-blocking device (VC-01, NCT02239354) and transplanted subcutaneously in the omentum (10). This trial however, faced inconsistent cell survival due to foreign body reaction to the encapsulation device (10), a phenomenon caused primarily by inflammation and excessive collagen deposition, and exacerbated by hypoxia (11, 12). These results brought attention to the importance of delivery methods and post-transplant environment on therapeutic success. To address this challenge, the devices were modified to incorporate pores to allow vascularization by the host (VC-02, NCT03163511) and entered clinical trials in 2017, combined with immunosuppressive therapy (3). Results from this pioneering study in humans showed beta cell formation in 63% of recipients, and circulating endogenous C-peptide in 35% of the recipients after 12 months, without teratoma formation (6). The level of secreted C-peptide was 20–40 pM in 33% (5/15) of recipients and <10 pM in all other recipients (3), which did not reach levels required for metabolic significance (≥100 pM at fasting) (5, 13) or insulin independence (≥500 pM at fasting) (14). To increase C-peptide levels, larger beta cell number, more devices per recipient, and higher pore density per device to increase vessel ingrowth were tested, achieving C-peptide levels > 30–230 pM in 60% (6/10) of the recipients, with non-detectible levels in others after 12 months (4). The lack of therapeutic response was attributed to PEC’s developmental potential towards alpha cells at the expense of beta cells and inefficient engraftment, resulting in cell loss, marked by acellular regions in all grafts, which cumulatively led to insufficient beta cell content (3). Moreover, 33.7% of recipients exhibited side effects of the immune suppression regimen and dropped out of the trial (3, 4, 6). The results of these trials highlighted the need for new strategies to improve the cell product, enhance graft vascularization, and reduce or eliminate immunosuppression as the next required steps. As an improved cell product, SC-islets [with 40-60% beta cell content (9)] entered clinical trials in 2021 (Vertex, NCT04786262) via infusion of 0.8 x 10^9^ cells into the portal vein, a similar approach used for donor-derived islet transplantations (15, 16). This resulted in robust C-peptide secretion detected 90 days post-transplantation (125 pM at basal, 424 pM post-meal), and after 1 year, 10 out of 12 recipients were insulin independent (442 pM C-peptide at basal, and 1274 pM post-meal) (15, 16). It is likely that similar to donor islets, SC-islets infused into the portal vein undergo instant and gradual attrition due to instant blood-mediated inflammatory response (IBMIR) and chronic inflammation as well as other factors, leading to the loss of insulin independence after 2.1 years and full graft loss after 5.5 years, despite immunosuppression (8, 17, 18). Thus, examining alternative transplantation sites, mainly intramuscular or intraperitoneal sites is an active area of research. In another recent trial (China, ChiCTR2300072200), the SC-islets developed from the differentiation of chemically induced pluripotent stem cells (ciPSC-islets with ~50-70% beta cells) were transplanted under the abdominal rectal sheath of one patient who was already immunosuppressed due to prior organ transplantation (19). Endogenous C-peptide was observed after 75 days, at 589 pM at fasting, leading to insulin independence, and reached 720 pM after 1 year (19). It is noteworthy that in this trial, ~20,000 IEQ/Kg ciPSC-islets were used, which is higher than that used in allogenic islets (~11,000 IEQ/Kg) and the SC-islets (~7,000 IEQ/Kg) infused into the portal vein, but lower doses of ciPSC islets could also be effective, which is not tested yet. In less successful clinical trials, intraperitoneal transplantation of encapsulated SC-islets was tested (VX-264, NCT05791201) using immunoprotective devices designed to eliminate the need for immunosuppression. However, insufficient C-peptide secretion led to the discontinuation of this trial (20). Results from VX-264 as well as the earlier trial, VC-01, strongly suggest that the use of immune-blocking devices, which are known for preventing inadequate vascularization, at least in the subcutis, compromises therapeutic success. Cumulatively, results from human trials highlighted the promise of SC-islets in achieving insulin independence in people living with T1D. The next steps are improving scalability and dosing, eliminating immune suppression, assessing short-term and long-term outcomes in various transplant sites, and developing methods to monitor the grafts post-transplant. To address these, ongoing research focuses on understanding and enhancing beta cell commitment and maturation, both of which are essential for improving the scalability of cell production and enabling more accurate prediction of insulin secretion per dose. Incorporating vascularization strategies is proven to promote graft survival, accelerate cell maturation, and overall enhance and sustain function, which could be a beneficial next step. Importantly, mitigating immunosuppression without blocking vascularization is an essential next step, which could be achieved via the generation of hypoimmunogenic SC-islets or engineering an immunomodulatory transplantation microenvironment. Developing methods to monitor the grafts *in vivo* and conducting head-to-head comparisons of transplant outcomes across various sites in research settings could also help identify optimal conditions for long-term efficacy with potential for clinical translation. In this review, we discuss recent advances in these three domains: cell production, vascularization, and immunomodulation as promising next steps for T1D treatment.

**Table 1:** Comparison of the clinical allogenic islet transplantation to SC-islets or PECs clinical trials.

Trial ID and country	Canada, France, Germany, Italy, Netherlands, Norway, Switzerland, United Kingdom, United States America: NCT04786262 (VX-880 or Zimislecel) EU: 2024-513929-23-00	China: ChiCTR2300072200	USA, Canada, Belgium NCT03163511 (PEC-01 in VC02)	Canada: Clinical human native islet transplantation
Cell type	SC-islets	ciPSC-islets	PEC	Human isolated islets
Cell number	0.8 x 10^9^ (cohort A, in two half doses one year apart, or cohorts B and C, one full dose [Mean weight 74 kg, thus ~7,000 IEQ) (2)]	1,488,283 IEQ (weight 75 kg, thus 19,843 IEQ/Kg)	250–500 x 10^6^ PEC, equivalent of 5,300 IEQ/Kg [cohort 1 (3)], and 2-3x higher [cohort 2 (4)]	11000–18600 IEQ/Kg Sustained survival: 15900 Non-sustained survival: 11900
Differentiation protocol	Pagliuca, 2014, PMID: 25303535, Veres, 2020, PMID: 32382023	Du, 2022, PMID: 35115708	Kroon, 2008, PMID: 18288110 Rezania 2012, PMID: 22740171	Primary tissue
% Beta cells at the time of transplant	~44% (as reported in Veres et al)	~56-72%	0%	N/A
Site of transplant	Portal vein	Under abdominal rectal sheath (intramuscular)	Abdominal subcutis	Portal vein
Encapsulation	No	No	Yes	No
Immunosuppression	Yes	Yes	Yes	Yes
Number of patients	2 (Half dose) + 12 (Full dose)	1	17	255
First C-peptide detection as reported	Day 90-365 (freedom from severe hypoglycemic events)	Day 14-75 (meal responsiveness Day 56)	Month 6 (in 35% of recipients)	Day 180
Fasting Cpeptide	125 pmol/L (day 90) 424 pmol/L (day 365)	102 pmol/L (day 7) 589 pmol/L (day 75) 722 pmol/L (day 365)	0–30 pmol/L	20 pmol/L
Post-meal C-peptide	Cohorts B and C: 424 pmol/L (day 90) 1274 pmol/L (day 365) 70–180 mg/dL (70% of time)	350.9 pmol/L (day 56) 834.1 pmol/L (day 120)	30–70 pmol/L (day 90) 30–170 pmol/L (day 180) 30–230 pmol/L (day 365)	600 pmol/L (day 365)
Available follow-up	90–365 days	75–365 days	365 days	Median 7.4 years (up to 20 years)
Insulin independence	10/12 (91.6%)	1/1 (100%)	0/17 (0%)	79% (yr 1), 32% (yr 5), 20% (yr 10), 8% (yr 20).
Time to insulin independence	Between 180–365 days	75 days	Not achieved	Median time: 95 days
Reduced insulin needs	Yes	Yes	No	Yes
Patient ethnicity Patient gender	White Female (5) and male (6)	Asian Female	Predominantly white Female and male	85% White, 13% Black, 1% Asian Canadian, 1% Indigenous Canadian. Female and male
Side effects	Neutropenia in 3 recipients, kidney injury in 2 participants. (Note: 2 deaths, one after 19.5 months due to cryptococcal meningitis because of immunosuppression, one at 30 months due to progression of preexisting neurocognitive impairment).	Adverse events limited to peri-transplantation period: pain at the puncture area, nausea and vomiting	Mild effects due to implant or explant procedure and VC-02 in 27.9% of recipients, and immunosuppression related effects such as uterine leiomyoma, parvovirus B19 infection, liver abscess, colitis, and pleural effusion in 33.7% of recipients	Reviewed in (7)
Reference	PMID: 40544428	PMID: 39326417	PMID: 35028608 PMID: 34861146 PMID: 38012450	PMID: 35588757

## 2 Summary of progress in hPSC differentiation towards beta cell fate

**Figure 1:**
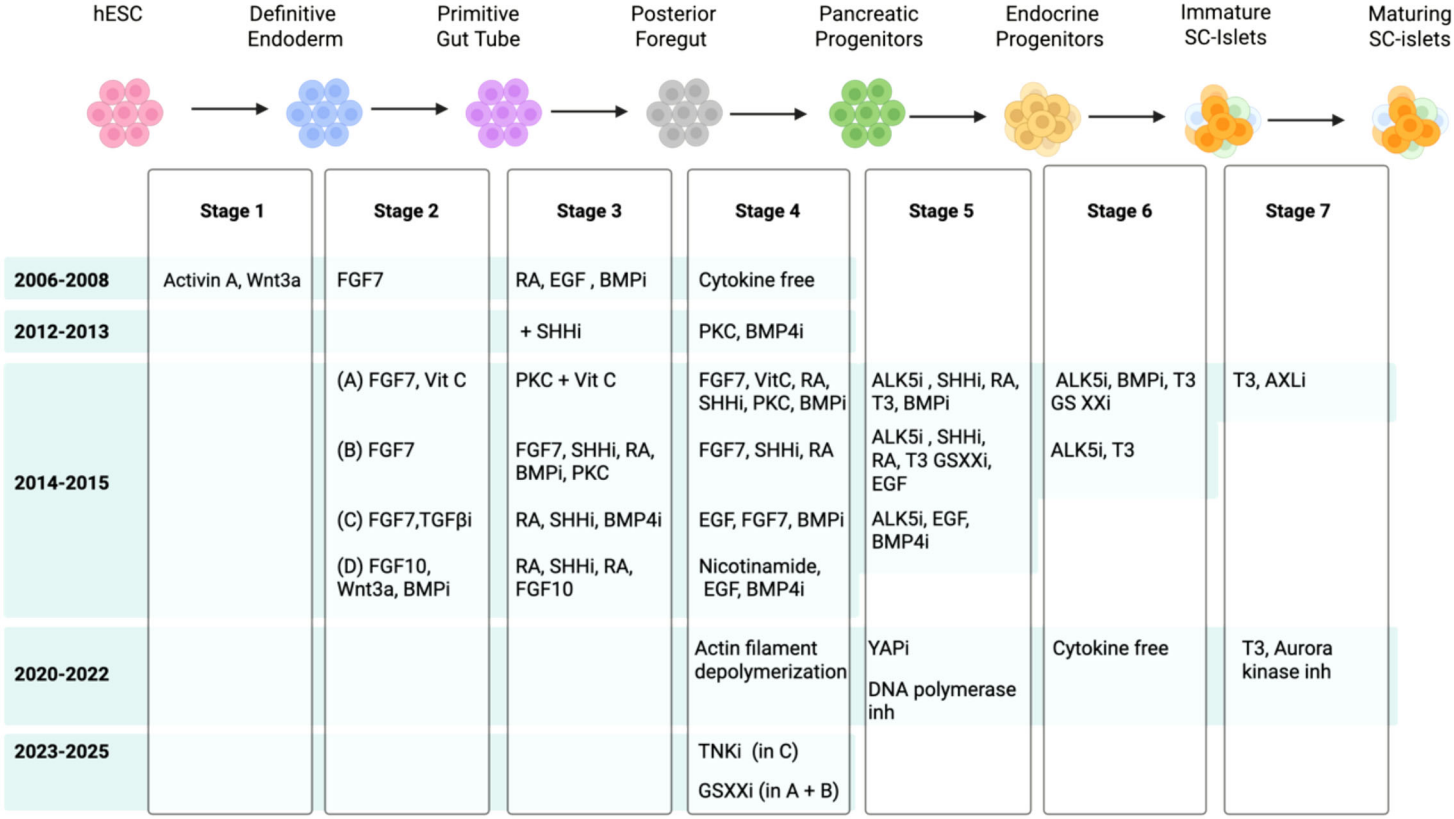
Schematic showing the summary of signaling pathways targeted to guide the stepwise differentiation of hPSCs to beta cells over the past 20 years (i is inhibition).

Research over the past decade yielded an enriched pancreatic progenitor population and promoted their developmental potential towards beta cell fate. The protocols generated vary depending on the stem cell line and culture conditions. The most prominent protocols are optimized for hESC lines H1, HUES8, MEL1, and CyT49, and their derivative reporter lines, as monolayers (21–24) or suspension aggregates (25–29). Significant progress has been achieved in the past few years in modulating multiple signaling pathways at each stage of differentiation to refine cell fate commitment, improve cell survival long-term in culture, and generate functional cells (**Figure 1**), which we discuss below.

### 2.1 Stages 1 to 4: commitment to pancreatic progenitors

Despite modifications, the principal signaling pathways targeted in all lines and various protocols are mostly similar. At stage 1 (definitive endoderm), activation of Nodal signaling (Activin A) (30–32) is used to mimic native development in which cells exposed to a high Nodal pattern to the anterior definitive endoderm and later give rise to the pancreas (33, 34). CD177 was identified as a cell surface marker of definitive endodermal cells produced at stage 1 of differentiation; thus, cell sorting to enrich the CD177^+^ population at this early stage boosts their differentiation towards the pancreas at later stages (35). Stages 2–3 mimic the primitive gut tube formation during native development and its patterning to the posterior hindgut and foregut, based on gradient exposure to low, medium, and high FGF, respectively. Moreover, two key factors for the induction of PDX1^+^ endoderm are retinoic acid (RA) and inhibition of sonic hedgehog (SHH). Studies in mouse development demonstrated that RA secreted from the mesoderm and SHH inhibition by the notochord induce PDX1 in the posterior foregut (36, 37). Thus, to induce PDX1 expression *in vitro* at stage 3, posterior foregut cells are exposed to RA and SHH inhibitor (Cyclopamine or Sant-1) to PDX1^+^ posterior foregut (38, 39). Additionally, in H1 and H9 hESC lines, the inhibition of BMP4 at stage 2 (in addition to FGF10) and stage 3 (in addition to RA, and inhibition of SHH) is also required to promote a pancreatic fate, without which the liver marker ALB is induced (31). Studies in mice demonstrated that BMPs regulate hepatic versus pancreatic fate decisions, and BMP2 and BMP4 signaling from the mesenchyme can promote a hepatic cell fate instead of PDX1^+^ pancreatic cells (40, 41). However, in the MEL1 line, BMP inhibition during stage 3 promoted the precocious induction of endocrine differentiation in PDX1^+^ pancreatic progenitors, generating polyhormonal cells that give rise to alpha cells after transplantation in mice (42), suggesting line-specific differences. In differentiating cells from the H1 line, adding vitamin C at stages 3 and 4 prevented the premature formation of endocrine cells (43). The combination of vitamin C and ALK5 inhibitor (RepSox) was shown to inhibit glucagon expression (44), and vitamin C’s roles in reducing oxidative stress and achieving epigenetic stability were speculated to be involved at this stage (45). Commitment of the PDX1^+^ cells to pancreatic progenitors that co-express NKX6–1 was first achieved by PKC activation and inhibition of BMP and ALK5, leading to simultaneous reduction of CDX2 (intestinal marker) and ALB (liver marker) (30). Experimentally in the H1 line, sodium bicarbonate supplementation during stages 1 and 2, and TGFβR1 at stage 4, also promoted pancreatic progenitor commitment, but reduced future commitment to ductal, acinar, epsilon, and gamma cells, thus propagating the cells towards beta and alpha cell fates (46). Later, it was shown that a combination of canonical Nicotinamide, BMP inhibition, and EGF treatment (upstream of PKC) at stage 4 robustly induces NKX6-1/PDX1 expression (47). Head-to-head comparison of stages 1 to 4 protocols using an NKX6-1-GFP iPSC line demonstrated that the use of Nicotinamide combined with EGF and BMP4 inhibition, induces NKX6–1 more robustly (71.3%), compared to combination of EGF, RA, and FGF7 (61.7%) or combination of PKC, RA, FGF7 treatment, with inhibition of BMP4 and SHH (56.3%) (48). Significant efforts continue to focus on understanding the signaling pathways that give rise to NKX6-1^+^ pancreatic progenitors. Recently, it was shown that effective inhibition of canonical Wnt signaling, by replacing Nicotinamide with a Tankyrase inhibitor, yields NKX6-1^+^ pancreatic progenitors with higher expression of integrins and less proliferative capacity, which propagated their future commitment to functional beta cells (21). These protocols have led to robust generation of pancreatic progenitors efficiently and at high numbers. However, the pancreatic progenitors are multi-potent and express markers such as SOX9 and GP2, which later become restricted to ductal and acinar cells, respectively, while NKX6–1 remains expressed in beta cells (49, 50). This multipotent potential leads to the formation of endocrine, ductal, and acinar cells when transplanted *in vivo* (21, 51), which challenges their efficacy for use in beta cell replacement therapies.

### 2.2 Stage 5: induction of endocrine cell fate

While stages 1 to 4 of differentiation model native developmental events, pathways governing endocrine, ductal, and acinar cell differentiation were identified via studying the developmental potential of pancreatic progenitors *in vitro* and *in vivo*. Imperial compound testing and systematic dose and exposure time optimization were used to develop protocols to induce endocrine (stage 5) and hormone^+^ (stage 6) cell fates (43, 52), while single-cell sequencing and pseudo time trajectory analysis of pancreatic progenitors in the form of organoids or after transplantation identified methods to induce acinar or ductal cell fates (53, 54). As a result, commitment to endocrine and hormone^+^ cell fates (stages 5-6) are the rate-limiting steps beta cell differentiation, due to significant cell death, and suboptimal or unsynchronized cell commitment, which is contrary to stages 1–4 which yield a high number of cells and up to 95% cell commitment. In addition, as endocrine committed cells are not proliferative, the endocrine or hormone^+^ populations cannot be expanded, contrary to acinar and ductal cells (55, 56).

In the native pancreas, NGN3 is transiently induced by Notch inhibition, which is sufficient for the induction of endocrine cell fate and formation of nascent islets (57, 58). However, during *in vitro* endocrine commitment (stage 5), inhibition of TGFβR1 and SHH, combined with low-dose RA and thyroid hormone (T3 or its analogue, GC1), with or without BMP inhibition, is required in addition to Notch inhibition (γ-secretase inhibitor XXi) (43, 52). Recently, Notch inhibition has been applied as early as stage 4 to increase the yield of endocrine commitment (2, 24, 45). It is noteworthy that pancreatic progenitor exposure to forskolin induced an acinar cell fate (53), while exposure to FGFs (FGF10 and FGF7) and CDK inhibitors promoted a ductal phenotype (54). In the developing pancreas, cell fate bifurcation is achieved via drastic cell migration, where acinar cells migrate to the tip, and ductal and endocrine cells migrate to the trunk of the branching pancreas tree. The NGN3 endocrine cells then undergo cell cycle arrest, delamination to dissociate from the ducts, and further egress from the branches (57). *In vitro* cytoskeletal modifications, such as actin filament depolymerization (59) or YAP signaling inhibition (verteporfin) (60), also enhance endocrine commitment. Interestingly, exposure to extracellular matrix components (ECM), such as fibronectin or vitronectin, promotes cell spreading, leading to a ductal phenotype, while laminin or collagen induces cell confinement, favoring endocrine differentiation (61). Pursuant to NGN3 expression, other endocrine markers such as NKX2–2 and Chromogranin A, and NEUROD1 are upregulated (in humans), and unlike NGN3, their expression is sustained in all endocrine cells in culture.

### 2.3 Stage 6 and onwards: induction of SC-islet formation

At stage 6, sustained inhibition of ALK5, γ-secretase, and BMP4, combined with T3, yields ~30–70% beta-like cell commitment depending on the protocol and cell line used (23, 24, 26, 29, 43, 52, 62). The remaining cells include alpha and delta cells, non-hormonal endocrine cells which are at an earlier developmental stage, and aberrant cell types such as polyhormonal cells (often C-peptide^+^/GCG^+^), and intestinal enterochromaffin-like cells (9, 63). Notably, a cytokine-free stage 6 protocol also produced SC-islets with a comparable beta cell content, suggesting that these compounds may not be essential for beta cell commitment at this stage and/or that the cell fates are pre-programmed at earlier stages (25). Intriguingly, protocols that promote alpha or delta cell fates have recently been developed where modifications are applied at earlier stages. To induce alpha cell commitment, transient BMP inhibition at stage 4 followed by sustained BMP inhibition at stage 5 (and elimination of FGF7, RA, and the inhibitor of SHH), generates NKX6-1^-^ pre-alpha cells, which, when treated with PdBu, a PKC activator, commit to alpha cell fate (GCG^+^/C-peptide^-^) (64). Delta cell differentiation can be achieved via modulations of FGF signaling, where prolonged FGF7 treatment during stage 5, combined with FGF2, induces SST^+^/HHEX^+^ delta cell commitment (65). The proportions of these populations vary significantly between hPSC lines and differentiation protocols, but beta-like cells often represent the largest fraction of SC-islets (25). In the native pancreas, ARX and PAX4 regulate alpha and beta cell fates, respectively, while loss of both PAX4 and ARX is associated with delta cell fate (66, 67). Supporting this, a study using PAX4/ARX double reporter iPSCs showed that sequential activation of PAX4 followed by ARX drives pancreatic endocrine commitment to alpha cells (68). Moreover, polarization of endocrine progenitors was shown to modulate ARX/PAX4 expression, where cAMP-induced polarization via EGR1 expression inhibited ARX expression and promoted beta cell fate, while lack of polarization led to continued ARX expression, promoting an alpha cell fate (69). Despite this progress, knowledge gaps exist in promoting a beta cell fate over other hormone-expressing cells *in vitro*. However, beta cell enrichment strategies are established, using CD49a^+^ (9), and it is noteworthy that the native human beta cells are enriched by positive selection using pan-endocrine marker HPi2^+^ cell selection, and further by negative selection for HPa3, which is expressed in all non-beta cells (70), while native mouse beta cells can be enriched by CD71^+^ cell sorting, indicating differences in cell marker expression between SC-derived versus native human or mouse islets (71). In addition, a recent study showed that cryopreservation of SC-islets enriches the beta cell population (29). The therapeutic efficacy of SC-islets versus pancreatic progenitors were first assessed by transplantation under the kidney capsule of streptozotocin (STZ)-induced hyperglycemic immunocompromised mice, where 2.5 x 10^6^ SC-islets (44% Insulin^+^/NKX6-1^+^) secreted ~ 4.6 ng/ml C-peptide after 16 weeks, 2-fold higher than that achieved with 5 x 10^6^ pancreatic progenitors after 21 weeks (43). Similarly, transplantation of 5 x 10^6^ HUES8-derived SC-islets (38% C-peptide^+^/NKX6-1^+^) under the kidney capsule prevented progression of hyperglycemia as early as 2 weeks post-transplant, and normalized glycemia after 12 weeks (52). Since these early studies, stage 6 protocols have been refined; however, *in vivo* function still largely depends on the cell dose and site of transplantation, with normalization of hyperglycemia typically requiring at least ~4 weeks of *in vivo* incubation (21, 25, 59). Furthermore, a few studies have tested an additional seventh stage of differentiation to induce glucose-responsive insulin secretion. R428, an inhibitor of AXL, used in combination with an ALK5 inhibitor, was shown to promote the expression of MAFA in beta-like cells 7–15 days post-treatment (43). Recently, ZM447439, an aurora kinase inhibitor, was added to stage 7 and improved beta cell maturation (23), while simultaneously reducing the proportion of enterochromaffin cells from 13% to 6.5% after 6 weeks of treatment (23). Consequently, transplantation of stage 7 SC-islets under the kidney capsule of mice resulted in detectable levels of human C-peptide after 1 month (23). Interestingly, the level of *in vivo* C-peptide can be increased by 2-fold if the SC-islets are maintained in culture for up to 6 weeks before transplantation, which continuously enhances maturation marker expression (23). High throughput single cell analysis of SC-beta cells generated by various protocols and their comparison to native fetal and adult human beta cells demonstrated that the SC-beta cells are transcriptionally more mature than fetal islets, but not as mature as the adult islets (63). This was due to the expression of gene networks associated with progenitor and neuronal fates in SC-islets, irrespective of the differentiation protocol (63). While the SC-islets secrete similar levels of insulin to native adult human islets *in vitro*, they have relatively lower insulin content, lower calcium influx, and immature insulin granules. A recent study showed that post-transplant in mice, the SC-islets continue to mature up to 4 months post-transplant, during which they gain increased MAFA expression, leading to an increase in insulin content and secretion, rather than changes in cell number or SC-islet composition over time, confirming that *in vivo* transplantation is required for beta cell functional maturation (72). This was also confirmed in human clinical trials, where transplanted SC-islets secrete higher levels of insulin over time, reaching a 3-fold increase after 1 year compared to 3 months (2), suggesting gradual maturation. Importantly, *in vitro*, the SC-beta cells secrete insulin in response to aberrant stimuli such as pyruvate, a phenotype corrected after 4 months of transplantation (72). Strategies tested to improve beta cell function include cell cluster re-aggregation (61), circadian clock entrainment (73), and inhibition of cell proliferation and cell cycle progression (74), some of which progressively improved glycolysis and TCA cycle-related gene expression, leading to glucose-stimulated insulin secretion, but do not achieve an adult phenotype. Thus, improving beta cell commitment and maturation before transplantation could not only accelerate therapeutic efficacy, and potentially lower the cell dose per person to achieve insulin independence.

## 3 The role of vascularization in improving cell therapies

**Figure 2:**
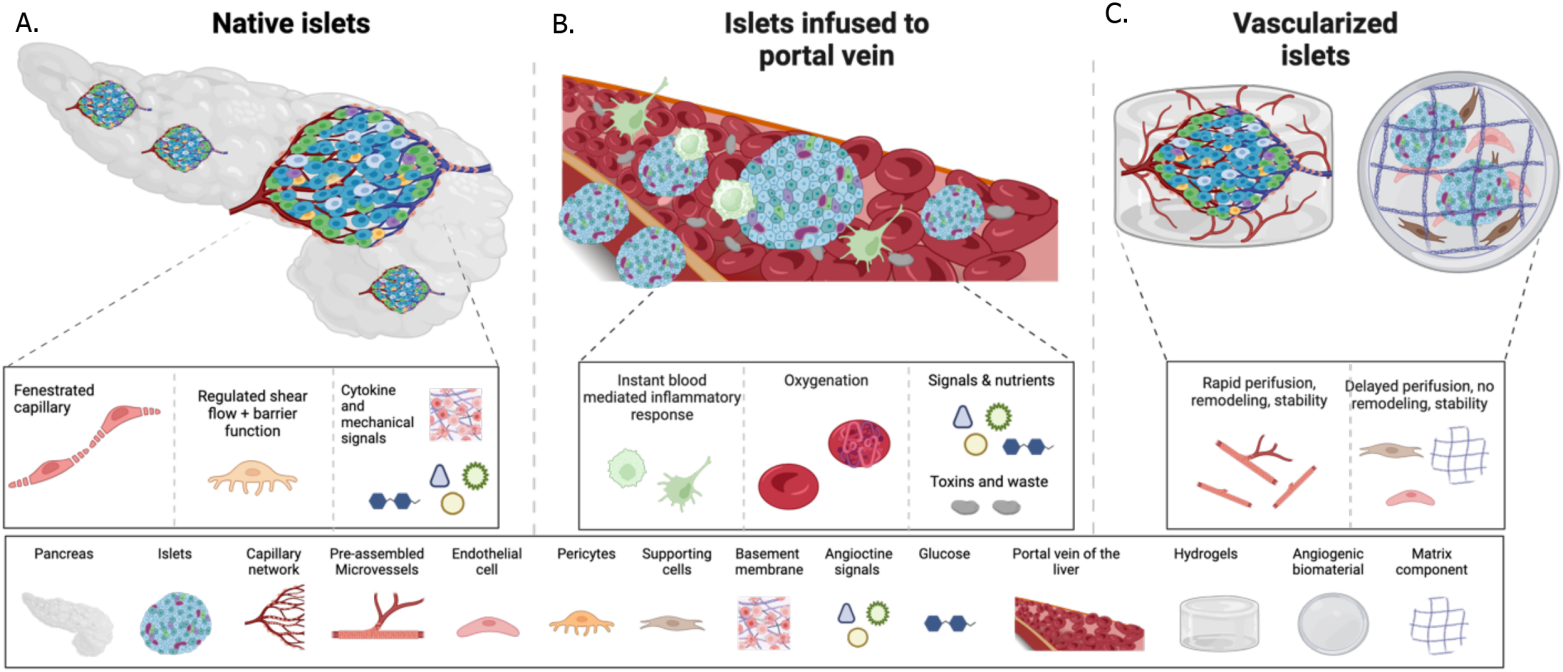
Schematic of islet connection to vessels in the native pancreas, versus post-transplantation into the portal vein naked, or the in the subcutis combined with vascularization interventions. **(A)** in the native pancreas islets are in close contact with the vascular cells, such as endothelial cells and pericytes secrete angiocrine signals and ECM that regulate the islet niche during development, health and disease, and form fenestrated vessels to accommodate rapid exchange of glucose and insulin. **(B)** A successful method of islet transplantation is via infusion into the portal vein. Initial contact with the host’s blood flow leads to rapid islet death due to instant inflammation. The surviving islets seed into the sinusoids and via this connect can sense glucose and secrete insulin into the bloodstream. **(C)** Vascularization strategies aim at pre or co-transplantation of vessels with the islets to recreate the islet vascular niche and accommodate endocrine function. Methods such as angiogenic biomaterials, cells and assembled vessels can be successful at various rates.

One of the challenges facing beta cell replacement therapies is the poor survival of the cells post-transplantation. The native islets are one of the most vascularized tissues in the human body (**Figure 2A**). Islet vasculature enables fine-tuned insulin response and plays a significant role in beta cell development and function, with islet vascular damage recognized as a major contributor to the initiation or progression of diabetes (75, 76). However, after isolation of islets from the pancreas, the basement membrane and the vascular network are digested. Thus, successful cell engraftment relies on a timely and robust connection with a new vasculature to ensure the delivery of nutrients, oxygen, and signaling molecules essential for cell survival. Graft vascularization is also imperative for glucose sensing and insulin secretion, both of which vary significantly depending on the transplantation site (**Figures 2B, C**), depending on the level of vascularization. Thus, site-specific and cell-type-specific vascularization strategies might be needed to promote cell survival.

### 3.1 Graft vascularization by the host at the site of transplant

Data obtained from PEC clinical trials indicated that although the encapsulation devices contain pores to allow vascularization, large regions within the grafts remain unvascularized, leading to significant cell loss (6). The issue of poor engraftment is also reported in the infusion of allogenic islets into the portal vein, where most islets face acute destruction by the IBMIR (77). Surviving islets are transported via portal vein circulation and distributed in the liver (78, 79), but only the islets that promptly lodge into liver sinusoids can survive and respond to glucose fluctuations by depositing insulin directly into the sinusoids (78, 79). Thus, connection with hepatic sinusoids is critical for the therapeutic efficacy of islet transplantation. Similar challenges are expected with SC-islets’ infusion into the portal vein. Interestingly, in a recent clinical trial, ciPSC-islets were transplanted in one patient intramuscularly under the highly vascularized rectal sheath, and no loss of cell mass was detected with MRI at the 1-year timepoint (19). It remains to be established if the intramuscular site is a privileged site for cell engraftment or if other factors, such as high cell dose or low MRI resolution, influenced the observation of no cell loss (19). An insightful study compared the therapeutic success of human or murine islet transplantation in various sites in mice: the portal vein, quadriceps muscle, or under the kidney, liver, or spleen capsules (80). Results demonstrated that normalization of blood glucose had a 100% success rate when islets were transplanted into the kidney capsule, 70% in muscle, 60% in the portal vein, 29% in the spleen capsule, and 0% in the liver capsule (80), suggesting that muscle could be a favorable site.

### 3.2 Vascularization strategies

Experimental models indicated that the vascular density of the transplantation site is directly linked to the therapeutic success. Transplantation of islets in highly vascularized sites in mice, such as under the kidney capsule, the anterior chamber of the eye (81), epididymal or mammary fat (82), and the cranium (83), achieved therapeutic success, unlike in the poorly vascularized subcutis, which does not support engraftment or endocrine function (51, 84, 85). However, the vascular density of the subcutis was enhanced via transplantation of a nylon mesh, which triggered angiogenic sprouting at the site of transplantation (86). The mesh was then removed, leaving a vascularized pouch in the subcutis where the islets or PECs were transplanted (device-less, DL). Consequently, engraftment was enhanced, leading to normalization of blood glucose in diabetic mice within ~25–30 days with 500 syngeneic mouse islets or 2000 human islet equivalents (IEQ) (86) or, within ~100 days, with 5 x 10^6^ hPSC-derived PECs (84). These novel studies confirmed that vascularization correlates with therapeutic success. A similar concept was then tested using silicon tubes, instead of nylon meshes, coated with angiogenic biomaterials (85). These methods, however, require multiple transplantations (insertion of meshes/tubes and removal, insertion of cells), and could also trigger a foreign body response, which could be detrimental to the grafts (10). Vasculogenic biomaterials have also been developed such as proteolytically degradable gels (PEG) conjugated to VEGF (PEG-VEGF) as vehicles for the delivery of syngeneic mice islets *in vivo* and achieved glycemic control with 200 syngeneic mice islets transplanted in the epididymal fat pad within 2 weeks, however this was not replicated when cells were transplanted subcutaneously (82). To further enhance vascularization, a combination of biomaterials and vascular cells has been implemented. For example, human umbilical vein endothelial cells (HUVECs) embedded in collagen cylinders, termed endothelialized modules, successfully vascularized the subcutis in diabetic mice and rats, and when co-transplanted with 750 rat islets, normalized glycemia in ~2 weeks (87). In more elaborate models, supporting cells such as human mesenchymal stromal cells (hMSCs) were co-seeded with HUVECs or iPSC-endothelial cells in meshes in the shape of vessels coated with fibrin matrix (88), forming vascularized meshes. When 500 rat islets were seeded on vascularized meshes and transplanted subcutaneously into diabetic mice, blood glucose normalization was achieved within ~2 weeks (88). The combination of the mesh and the hMSCs supported vessel integrity and led to retention for 100 days as tested (88). Overall, biomaterials-based studies successfully demonstrated that by vascularizing the subcutis, blood glucose normalization can be achieved. Long-term studies are needed to assess biomaterial stability *in vivo* and ensure that they do not get digested or damaged with time. An overall challenge is that the predetermined size and shape of the biomaterials dictate network size, vessel diameter, and prevent vascular remodeling. Vessel remodeling, also referred to as angio-adaptability, is the driver of tissue development, expansion, and cell fate commitment (89). As hPSC-derived cells also often transition through cell fates (for example, from PECs to islet cells, or SC-islets to mature islets), they might require a certain level of angio-adaptability to meet hemodynamic inputs, metabolic and developmental needs, and accommodate endocrine function (89, 90). Thus, cell-based vascularization strategies that can give rise to a vessel network with angio-adaptability could be most beneficial post-transplant. We previously showed that using intact microvascular fragments, which are in vessel shape and do not need single-cell assembly, can achieve normoglycemia within the first week of subcutaneous transplantation (51). The microvascular fragments were isolated from the adipose tissue (human or rat) while retaining intact endothelial lumen and pericyte coverage at a physiological ratio (91), and secrete extracellular matrix (92) and angiocrine signals (93, 94) that support sprouting angiogenesis *in vitro* (91) and *in vivo* in the subcutis (51). The microvessels not only alleviated hypoxia in co-transplanted islets or pancreatic progenitors but also developed a robust exchange interface between the host and the graft, improving glucose sensing and insulin secretion (51). Therefore, normalization of blood glucose was achieved as soon as the beta cells were developed from pancreatic progenitors, or immediately with human islets at the subtherapeutic dose of 1500 IEQ, were transplanted subcutaneously in STZ-induced or NRG-Akita diabetic mice (51). This strategy was compared to single HUVECs, which formed leaky and unstable vessels without therapeutic advantage, demonstrating the role of perivascular cells such as pericytes in forming vessels with appropriate integrity and function (51). Others showed that *in vitro* co-culture of microvessels with mouse islets significantly improved viability by reducing intracellular reactive oxygen species (ROS) levels and upregulating ROS-scavenger enzymes in beta cells (95). Moreover, insulin secreted by beta cells binds to insulin receptors and insulin-like growth factor receptors (IGFR) on endothelial cells, enhancing vessel sprouting (96). Co-transplantation of 250 mouse islets with microvessels under the kidney capsule lead to normoglycemia in 87.5% of transplanted mice by day 28 post-transplant, compared to 12.5% of mice who received islets alone (97), and increasing the islet number to 500 or 1000 restored normoglycemia in 100% of mice when microvessels were co-transplanted, but not without them (97).

## 4 Reducing immune suppression regimen post-transplantation

**Figure 3:**
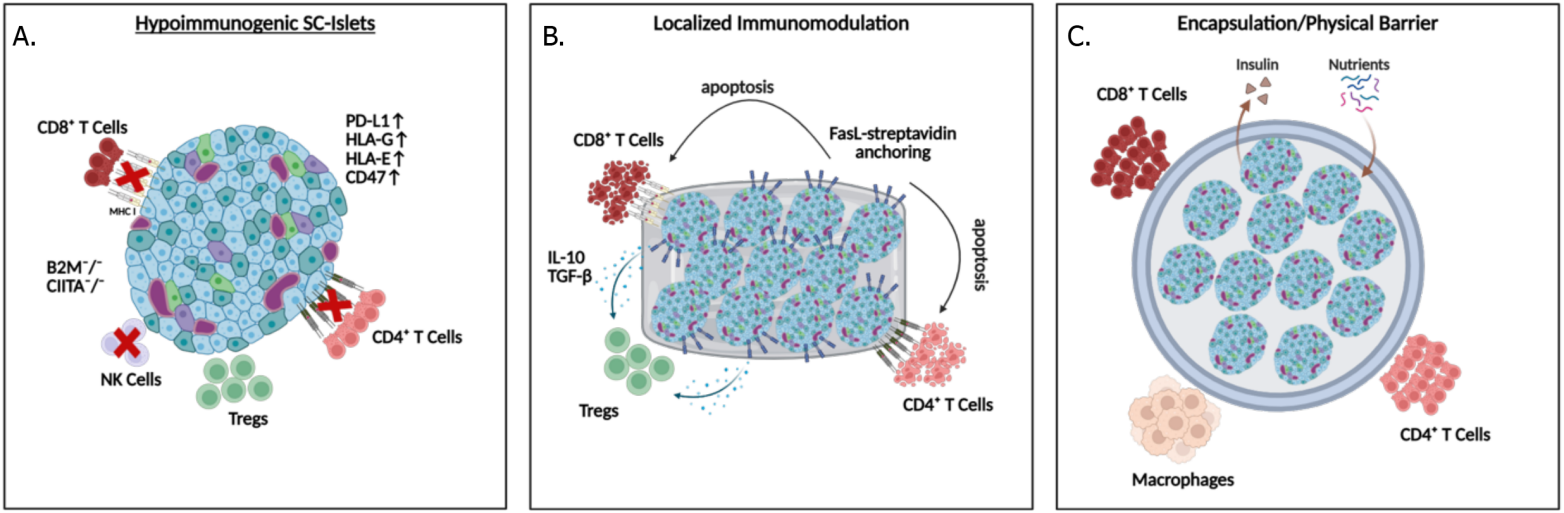
Strategies to protect SC-islet grafts from immune rejection: **(A)** Hypoimmunogenic islets can be generated with ablation of HLA expression while maintaining HLA-G and E, and overexpression of PD-L1, or CD47, **(B)** localized immunomodulation by delivering FasL, IL-10 and TGFβ to the transplantation environment, and **(C)** physical barriers can be applied to prevent direct contact between immune cells and the transplanted islets.

An overriding challenge in beta cell replacement therapy is the need for lifelong immune suppression to prevent graft rejection, which can cause significant health complications (98–100). Reports from clinical trials demonstrated adverse effects of immunosuppression in 33.7% of recipients (3, 6), and children are not recommended to undergo immunosuppression regimens, hence limiting the use of cell therapies for a group most affected by T1D. Importantly a 20-year report of a Canadian islet transplantation center showed that despite receiving immunosuppression that mitigates T cell rejection, the median graft survival is 5.9 years after infusion into the portal vein (8). Moreover, a multi-center report (39 centers and 1210 islet recipients) demonstrated that initial engraftment in the liver, which is challenged by IBMIR, is completely independent of long-term islet loss (98). These reports highlight the role of non-T cell immune mechanisms in the portal vein that can jeopardize therapeutic longevity. As such, immune B cells can also contribute to graft rejection by producing donor-specific antibodies, which correlates with poorer graft survival in humanized allogeneic models, emphasizing the importance of developing strategies that minimize B cell activation and antibody production (101, 102). Non-specific immune mechanisms such as inflammation, hypoxia-induced cytokine production, and thrombotic reactions can also cause acute or progressive islet loss (103, 104). Pro-inflammatory cytokines such as IL-1β, IFN-γ, and TNF-α are often released by infiltrating immune cells, which promotes beta-cell apoptosis (105, 106), or increases T cells and macrophage recruitment to the transplant sites (107). On the other hand, anti-inflammatory cytokine IL-10 can promote graft survival by modulating the innate immune response, as demonstrated by administration of an IL-1 receptor antagonist (anakinra) alongside a TNF inhibitor (etanercept) at the time of transplantation (8, 108). Moreover, in response to hypoxia, the transplanted islets can secrete inflammatory cytokines, propelling their destruction (105). This highlights the importance of modulating both innate and adaptive immune attacks in the absence of systemic immunosuppression. Below, we discuss strategies to overcome immune rejection (**Figure 3**).

### 4.1 Encapsulation and physical barriers

Encapsulation involves surrounding the islets with a semipermeable membrane that allows the exchange of nutrients, oxygen, and insulin while preventing the infiltration of immune cells (109). Recent advancements in biomaterial engineering have focused on improving biocompatibility and reducing fibrosis, which has historically limited the effectiveness of this approach (110). However, the inability of SC-islets to engraft, function, or mature *in vivo* due to incomplete interaction with the environment remains a significant drawback. Combining encapsulation with localized delivery of immunosuppressive agents has shown potential in enhancing the long-term survival of islet grafts (111).

### 4.2 Hypoimmunogenic SC-islets

Hypoimmunogenic hPSCs are engineered to have reduced immunogenicity. The most common strategy involves the deletion or downregulation of human leukocyte antigens (HLAs), which present the antigens to T cells, thus their ablation eliminates T cell-mediated rejection (112). This can be overcome by targeting key regulator of HLA Class I, beta-2 microglobulin (B2M), and a master regulator of HLA Class II, class II transactivator (CIITA). Deletion of all HLAs, including HLA-A, B, and C, from iPSCs provided initial protection from acute rejection after transplantation into allogeneic humanized NSG mice, but T cell-mediated rejection led to graft loss within 5–6 weeks post-transplantation (113). To mitigate this, additional genetic modifications such as CIITA knockouts, which delete HLA class II but retain HLA-G, improved immune evasion in a xenogeneic mouse model (114). Unlike total HLA ablation, CIITA knockout suppressed CD4^+^ T-cell responses while preserving some resistance to NK cell-mediated lysis, thereby extending graft survival (115). Additionally, overexpression of PD-L1, which binds to its receptor on T cells and reduces their cytotoxicity, and on Tregs, to recruit these immune-modulating cells to the graft, can create localized immune tolerance (116–118). Inducible PD-L1 expression under the insulin promoter improved early survival of SC-islets transplanted under the kidney capsule of NOD.HLA-A2.1 mice, with significantly higher survival at day 3 post-transplantation; however, by day 14, all grafts were lost, suggesting that PD-L1 alone offers only transient protection in an autoimmune setting (119). Combining PD-L1 with PD-L2 and HLA-G expression conferred robust resistance to both T cell and NK cell-mediated lysis, prolonging graft survival in humanized mouse models, highlighting the advantage of multi-gene immuno-evasive engineering (120). Supporting this, SC-islets engineered solely with PD-L1 were xenorejected in diabetic NOD mice after 2 weeks, but co-expression of immunomodulatory cytokines, IL-10, TGF-β, and IL-2, effectively promoted regulatory T cell recruitment, suppressed effector responses, and restored normoglycemia for up to 8 weeks (111). Another innovative approach involved the use of HLA-E overexpression to inhibit NK cell activity (115). NK cells are pivotal in immune rejection when HLA expression is absent. Retention of HLA-A2 in hPSCs restored HLA-E surface expression, which significantly reduced NK cell degranulation *in vitro* (115). When SC-islets were generated from these engineered hPSCs and transplanted into humanized NSG-MHC null mice, they survived for up to 16 weeks (115). Further developing these strategies, CD47, a cell surface protein that inhibits phagocytosis by macrophages and dendritic cells, has been introduced alongside HLA class I and II deletion (B2M-/- and CIITA -/-) (121). CD47 further acts as a compensatory signal for the loss of HLA Class I, which triggers a missing-self phenotype, thus indirectly inhibiting NK cell activation to evade both innate and adaptive immune responses (122–124). Further knockout of HLAs (B2M-/- and CIITA-/-) and overexpression of CD47 (called hypoimmune platform or HIP) led to loss of immunogenicity, via inhibition of macrophage and NK cell-mediated clearance, thus evading both innate and adaptive immune responses (122, 123). Of note, the immunomodulatory effect of CD47 is species-specific and dependent on tissue-appropriate expression levels, which are critical for effective suppression of NK cell activity and avoiding off-target immune activation (122, 123). The HIP engineering can be applied to both iPSC-derived and primary human islets as well as non-human primates. The HIP-engineered human donor islets survived in immunocompetent, allogenic diabetic mice, and HIP-engineered rhesus monkey islets survived for 40 weeks in rhesus monkey recipients without immunosuppression. Thus, HIP-engineered primary human islets are currently being tested in clinical trials, where cells are transplanted intramuscularly without immunosuppression (Sana Biotechnology, NCT06239636) (125). Although SC-islets have not been reported to form teratomas or tumors in transplantation models, unlike undifferentiated progenitors, maintaining the ability to eliminate grafted cells remains a crucial safety consideration. Accordingly, universal hypoimmunogenic lines are being increasingly engineered with features that enable controlled clearance of the graft if adverse events arise. These lines incorporate safety switches, harboring an inducible suicide gene system. For example, iCasp9 which is a pro-apoptotic system activated by a small molecule drug AP1903 (rimiducid), Herpes Simplex Virus thymidine kinase (HSV-TK) which converts the prodrug ganciclovir into a toxic nucleotide analog, resulting in DNA chain termination and targeted cell death, and other thymidine kinases that can also generate toxic analogs, thusenabling targeted ablation of the grafts and combining immune evasion with a robust safety mechanism (120, 123, 126–129).

### 4.3 Localized immunosuppression approaches

Localized immunosuppression represents a targeted strategy to mitigate the systemic side effects of traditional immunosuppressive drugs. By focusing on the delivery of immunosuppressive agents directly at the transplantation site, this approach reduces systemic toxicity while maintaining effective immune modulation. Studies have shown that presenting islet surfaces with Fas ligand (FasL) can effectively induce apoptosis in infiltrating Fas-expressing T cells, thereby reducing graft rejection in murine models (130). To implement this strategy, a streptavidin-based anchoring system has been used to transiently display FasL on the surface of SC-islets, promoting early systemic tolerance and long-term immune privilege. These findings show streptavidin-anchored FasL (SA-FasL) induced apoptosis in Fas-expressing immune cells, with tolerance maintenance dependent on CD4^+^CD25^+^Foxp3^+^ Tregs and phagocyte-derived TGF-β in fully immunocompetent C57BL/6 mice (130). In diabetic rhesus macaques, a non-human primate, co-transplantation of SA-FasL-conjugated biodegradable microgels with allogeneic islets under transient rapamycin therapy enabled normoglycemia and graft survival for over 6 months (131). Extending this strategy, immune-homeostatic microparticles co-presenting FasL and MCP-1 (a monocyte-attracting chemokine), loaded with the diabetes-associated autoantigen GAD524–543, were shown to induce T cell apoptosis and expand regulatory T cells in hyperglycemic NOD/ShiLtJ mice, preventing diabetes progression via antigen-specific immune tolerance (132). Nanoparticles can also deliver specific immunomodulatory molecules, such as CXCL10 inhibitors, directly to the graft site, to suppress T cell recruitment and mitigate inflammation, significantly enhancing graft survival (133). Adult porcine islets encapsulated in alginate microcapsules enriched with CXCL12 into the omental sac of diabetic and healthy non-human primates, without systemic immunosuppression, demonstrated a transient modulation of systemic cytokine responses, with 2- to 6-fold changes in key inflammatory markers during the first four weeks post-implantation (134). The study highlighted CXCL12’s potential to provide short-term immune shielding and support the potential of using localized immune modulation strategies (134). In addition, co-transplantation of islets with accessory cells that exert immunomodulatory characteristics, and at times, vasculogenic potential, has also been tested. Among these, three multipotent cell types: human bone marrow mesenchymal stromal cells (hMSC) (135), human umbilical vein perivascular cells (HUCPVC) (136), or human amniotic epithelial cells (hAEC) (137) showed the most promise. These cells secrete anti-inflammatory and angiogenic signals when cultured *in vitro*, which could create a favorable post-transplantation environment. While the exact mechanism by which they improve islet transplantation in mice is not teased out, their accessibility and potential to expand and store make them an attractive source for improving islet transplantation. Lineage tracing studies would be highly interesting to assess their fate post-transplantation, especially because these cells are not only multipotent but also highly proliferative, and therefore, their clinical safety should be assessed.

## 5 Conclusions and future directions

While the SC-islets have shown great promise to transform T1D treatment, but their clinical dissemination to many patients requires improving beta cell functional maturation, enhancing integration into the host post-transplant, and limiting the need for systemic immunosuppression. Recent advances have significantly improved the beta cell profile differentiated from hESCs, however, their functional and metabolic maturity requires prolonged *in vivo* incubation (23, 138, 139). While this by itself is not a dealbreaker, recent trials showed that increasing cell number could be significantly advantageous therapeutically (19). While tumor/teratoma formation from SC-isles have not been reported after transplantation into mice, persistent presence of non-endocrine cells, even those from mesoderm lineage, has been reported by many groups, suggesting that increasing cell number to compensate for low insulin content/secretion of SC-beta cells could lead to adverse effects (9, 138, 139). Cell enrichment strategies are available yet significantly challenge scaling up (9, 35, 140). As a result, understanding pathways that lead to beta cell commitment specifically, and controlling the ratio of beta cells versus other cell types, and/or improving functional maturity of beta cells *in vitro* could be highly beneficial for clinical translation. Moreover, incorporating vascularization strategies to reduce cell loss post-transplantation could significantly improve clinical outcomes, however challenges in vascularization strategies are scaling up primary vessel sources, longevity of biomaterial-based strategies, and inability of single cell strategies to assemble into a functional and stable vessel network in a timely manner post-transplant. While many have warned that vascularization could increase immune cell delivery to the graft site, this appears contrary to vessel barrier which controls extravasation of immune cells. Thus, incorporating a functional vasculature could in fact prevent inflammation. However, research in this domain, as well as comparison of vascularization efficacy in various transplantation sites is highly needed. However, this is indeed in the context of immunosuppression, thus, incorporating innovative methods such as patient-derived cells/vessels, is also an important next step. While hypoimmunogenic SC-islets hold great promise for preventing graft destruction by T cells, combining them with immunomodulating strategies that prevent the recruitment of other immune cell types to the graft might be required. Moving forward, platforms that integrate lineage-optimized SC-islets, vascular-supportive niches, and engineered immune tolerance are likely to be the most effective path for large scale dissemination of beta cell replacement therapy.
